# Flavor Quality and Lipid-Lowering Function of Mixed Fermented Pu-erh Tea with Various Monascus Species

**DOI:** 10.3390/foods14111894

**Published:** 2025-05-26

**Authors:** Xiaomin Chen, Yao Hu, Zhen Zeng, Xu Zhang, Yahui Huang

**Affiliations:** 1 College of Horticulture, South China Agricultural University, Guangzhou 510000, China; chenxiaomin@stu.scau.edu.cn (X.C.); huyao202504@163.com (Y.H.); zzhen2004@126.com (Z.Z.); 2 College of Forestry and Landscape Architecture, South China Agricultural University, Guangzhou 510000, China

**Keywords:** monascus, Pu-erh tea, mixed fermentation, lipid reduction

## Abstract

As a potential raw material with a variety of bioactive compounds, Pu-erh raw tea can produce rich flavor and health benefits through natural fermentation or microbial fermentation in traditional processing. However, the traditional fermentation process has some problems such as a long fermentation period and unstable quality. Monascus, a kind of fungus used in both medicine and food, has been proved to have many beneficial effects such as lowering cholesterol and regulating blood lipids. Therefore, in this study we investigated whether the fermentation of mixed monascus could significantly improve the flavor quality and lipid-lowering activity of Pu-erh raw tea. We added four kinds of monascus to unfermented Pu-erh raw tea (UT) to obtain a fermented Pu-erh raw tea (FT). The quality of the two tea samples was determined and an in vitro lipid-lowering experiment was conducted. The results show that the contents of water extractives, flavone, trans-catechins (GCG and CG), theabrownins, and caffeine in FT are significantly higher than those in UT, increasing by 19.41%, 14.47%, 18.76%, 29.82%, and 10.67%, respectively. In terms of aroma, linalool was the characteristic compound of UT, presenting a floral note. D-Limonene was the key characteristic substance of FT, manifested as lemon, toast, and wood. In terms of taste, FT has a high content of bitter amino acids and caffeine, a low content of catechins, and is rich in carbohydrate substances, forming a characteristic mild and mellow slightly bitter taste with reduced astringency. In addition, the relative contents of active substances with lipid-lowering effects such as quercetin, quercitrin, ascorbic acid, and sorbitol in FT were higher than those in UT, increasing by 83.09%, 81.73%, 89.86%, and 92.76%, respectively. The effect of FT on regulating cellular lipid metabolism was superior to UT based on cell experiments. The research results provide a scientific basis for the deep processing and functional development of Pu-erh raw tea.

## 1. Introduction

Tea is one of the world’s three major non-alcoholic beverages. It originated in China and spread globally. As a perennial evergreen woody plant, the tender leaves and buds of the tea shrub (*Camellia sinensis*) can be processed into six major types of tea using different techniques—green tea, white tea, yellow tea, oolong tea, black tea, and dark tea. Many characteristic components in tea, such as polyphenols, caffeine, amino acids, and vitamins (A, B2, C, E, and K), have strong health benefits. Studies have shown that the active components in tea can reduce cell damage, lower the risk of cardiovascular diseases, and have health benefits such as anti-diabetic and anti-hyperlipidemia effects and the regulation of gastrointestinal flora [1]. Pu-erh tea is a kind of post-fermented dark tea that is a specialty of Yunnan Province, China. It is made using Yunnan large-leaf sun green tea as the raw material and special processing. Pu-erh tea undergoes a distinctive aging process, during which its flavor profile gradually transforms over time, developing greater depth and mellowness [2]. Pu-erh tea is categorized as Pu-erh raw tea or Pu-erh ripe tea; Pu-erh raw tea is green in color and astringent in taste, while Pu-erh ripe tea is artificially fermented, reddish brown in color, and mellow in taste [3].

Microbial fermentation plays an important role in the processing of Pu-erh tea and has a decisive influence on the quality and character formation of the tea [4]. The microorganisms involved in the fermentation of Pu-erh tea include *Aspergillus niger*, *Aspergillus oryzae*, *Penicillium*, *Saccharomyces*, and *Bacillus subtilis* [5,6,7,8,9]. Studies have shown that the exogenous addition of microorganisms can improve the growth environment of microorganisms and enhance the flavor quality of Pu-erh tea [10,11]. Although single-strain fermentation can improve some of the qualities of Pu-erh tea, mixed-strain fermentation has some advantages. The application of mixed-culture fermentation demonstrates significant improvements in the organoleptic properties of Pu-erh tea, notably by enriching its aromatic complexity and palate richness. In addition, fermentation with mixed strains can significantly shorten the fermentation time of Pu-erh tea. By artificially adding specific strains, the microbial community structure in the fermentation process can be better controlled, making the fermentation process more stable and controllable.

Red yeast rice, which is made via monascus fermentation with rice as the main raw material, is widely used in traditional food brewing, medical care, and other fields and has great development and research value. Monascus, the dominant strain in red yeast rice, is a traditionally used, small filamentous fungus with abundant species and numerous metabolites, including lovastatin, GABA, monascus pigment, and other active substances, which have beneficial effects such as lowering blood lipids and blood sugar as well as an anti-oxidation effect [12]. Monascus is widely used in the brewing industry, as a color additive, and in fermented foods, and many of the secondary metabolites it produces are beneficial to the human body [13,14,15]. In recent years, there have also been relevant reports on the fermentation of Pu-erh raw tea following monascus inoculation. Tian et al. [16] determined and analyzed the aroma of Pu-erh raw tea fermented by purple monascus, producing a tea with a rice-qu aroma, with 1,2-dimethoxybenzene, 1,2,3-trimethoxybenzene, and (E)-linalool oxide (pyranoid) the main aromatic substances. Deng et al. [17] used purple monascus to ferment Pu-erh raw tea and found that the resulting tea contained lovastatin, which had stronger hypolipidemic and anti-atherosclerosis effects and showed superior anti-inflammatory effects compared to Pu-erh raw tea.

In this study, four purple monascus strains were isolated and purified from commercial monascus rice and mixed into Pu-erh raw tea in equal proportions for fermentation. The flavor quality and lipid-lowering function of the tea samples before and after fermentation were measured and compared, and the effects of the mixed fermentation with various monascus strains on the flavor quality and health care efficacy of the Pu-erh raw tea were investigated. This study clarifies the effect of mixed monascus inoculation on the flavor quality of Pu-erh tea and provides a reference for monascus fermentation and the rich processing of Pu-erh tea.

## 2. Materials and Methods

### 2.1. Chemicals and Reagents

Epicatechin (EC), catechin (C), gallocatechin (GC), epigallocatechin (EGC), epigallocatechin gallate (EGCG), gallocatechin gallate (GCG), epicatechin gallate (ECG), catechin gallate (CG), theobromine, theophylline, and caffeine were all purchased from Sigma-Aldrich (USA). Methanol, isopropanol, and trifluoroacetic acid were all chromatogram-pure (Shanghai, China, Shanghai Aladdin Biochemical Technology Co., LTD.). Purity > 99%. Ninhydrin, stannous chloride, potassium dihydrogen phosphate, disodium hydrogen phosphate dodecahydrate, methanol, sodium carbonate, Folin’s phenol, boric acid, and sodium hydroxide were of analytical grade and purchased from Macklin Biochemical Technology Co., Ltd. (Shanghai, China). Phosphate-buffered solution (PBS), Fetal Calf Serum (FBS), oleic acid (OA), MTT, and DMSO were all purchased from Sigma-Aldrich (St. Louis, MO, USA). Potato Dextrose Agar (PDA), Malt Extract Agar (MEA), High-glucose medium (DMEM), and pancreatic enzymes were all purchased from Sangon Biotech (Shanghai, China).

### 2.2. Methods of Isolation and Identification of Monascus

Commercially available red yeast rice (Anhui Mengsheng Pharmaceutical Co., Ltd., Hefei, China) was crushed by a pulverizer and sieved with a 40-mesh sieve. Under the ultra-clean bench, 10 g of red yeast rice powder was taken and shaken evenly in a 90 mL sterile water triangle bottle with glass beads. After full shock, the liquid was diluted several times and 0.2 μL was absorbed and evenly coated on PDA medium, and cultured in a 30 °C biochemical incubator. When the colony entered the vigorous period, the mature red colony was picked with a sterile inoculation ring and streaked for 5 days. A single purified colony plate was picked again and streaked for 3–4 times to obtain a purified strain. The obtained strain was recorded with a number and stored in a 4 °C refrigerator. The isolated and purified monascus was seeded in Malt Extract Agar (MEA) medium to observe the colony morphology.

A suitable growing plate was taken to pick the mycelium, which was ground into powder with liquid nitrogen and added into a 1.5 mL centrifuge tube. Then, 400 μL of Buffer Digestion solution and 4 μL of β-mercaptoethanol were added and mixed by shock. The mixture was water-bathed at 65 °C for 1 h until the cells were completely lysed, mixed upside down every 10 min, and 200 μL Buffer PF was added. The mixture was thoroughly mixed upside down, refrigerated at −20 °C for 5 min, and centrifuged at room temperature at 10,000 rpm for 5 min, and the supernatant was transferred into a new 1.5 mL centrifuge tube. Isopropyl alcohol was added in equal volume, reversed 5 to 8 times to fully mix, left at room temperature for 2–3 min, and centrifuged at 10,000 rpm for 5 min, after which the supernatant was discarded. Then, 1 mL of 75% ethanol was added, reversed for 1–3 min, and centrifuged at 10,000 rpm for 2 min, and the supernatant was discarded. The lid was opened and inverted at room temperature for 5–10 min until the residual ethanol was completely volatilized. The obtained DNA was dissolved with 50–100 μL TE Buffer and amplified by polymerase chain reaction. The PCR-amplified products were verified by electrophoresis. The product was sent to Shanghai Shenggong for sequencing. The sequencing results were compared by BLAST 1.4.0 in the NCBI database.

### 2.3. Solid Fermentation

The isolated monascus was moved to the PDA slope and cultured at 28 °C for 10 days. The spores were washed off with 0.3% Tsum 80 and filtered with three layers of gauze to make a monascus solution with a spore concentration of 10^6^ CFU/mL. The Pu-erh raw tea was moistened to make its water content 50%, steamed under a high pressure of 103.4 kPa at 120 °C for 20 min, and then removed and spread to room temperature. The monascus solution was inoculated to the cooled tea leaves at a proportion of 10%, then evenly mixed into a bamboo basket and fermented for 28 days at a temperature of 28 °C and a humidity of 70–80%. During the process, an appropriate amount of water was added, the tea leaves were turned, and finally, they were dried at 65 °C for 4 h.

### 2.4. Determination of Main Biochemical Components

The moisture content was determined according to GB 5009.3-2016 [18]: a tea sample (2 g) was placed in an oven at 101~105 °C and dried for 4 h. After being taken out, it was cooled in a desiccator and weighed. The drying process is repeated until the mass difference between two consecutive weighings is ≤2 mg. The content of water extract was determined according to GBT 8305-2013 [19]: a tea sample (2 g) was added to 200 mL of boiling water and extracted in a 100 °C water bath for 45 min. The tea infusion was filtered under suction, and 50 mL of the filtrate was transferred to an evaporating dish. The solution was then evaporated to dryness in a water bath. The evaporating dish was placed in an oven at 103 ± 2 °C and dried to constant weight (mass difference between two consecutive weighings ≤ 0.001 g). Tea polyphenols were determined by GBT8313-2018 [20]: a tea sample (0.2 g) was added to 5 mL of 70% methanol and extracted at 70 °C for 10 min. After being cooled to room temperature, it was centrifuged at 3500 r/min for 10 min, and the supernatant was transferred to a 10 mL volumetric flask. The residue was re-extracted with 5 mL of 70% methanol, and the above steps were repeated. The combined extracts were diluted to 10 mL and mixed well for later use. From the above solution, 1 mL was taken and mixed with 5 mL of 10% Folin–Ciocalteu reagent, followed by a reaction time of 3–8 min. Then, 4 mL of 7.5% Na_2_CO_3_ solution was added, and the mixture was allowed to react for 1 h. The absorbance was measured at 765 nm wavelength using a 10 mm cuvette. Flavones were measured by the colorimetric method with aluminum trichloride: a tea sample (2 g) was added to 80 mL of boiling water and extracted in a 100 °C water bath for 30 min. The mixture was filtered under suction, and the volume was adjusted to 100 mL with water. From the resulting solution, 0.5 mL was taken and mixed with 10 mL of 1% AlCl_3_ aqueous solution. After 10 min, the absorbance was measured at 420 nm wavelength using a 10 mm cuvette. The total amount of free amino acids was determined by GBT8314-2013 [21]: a tea sample (3 g) was added to 450 mL of boiling water and extracted in a 100 °C water bath for 45 min. The mixture was filtered under suction, and the volume was adjusted to 500 mL with distilled water. From the resulting solution, 1 mL was aliquoted and mixed with 0.5 mL of phosphate-buffered solution, followed by the addition of 0.5 mL of 2% ninhydrin aqueous solution. The reaction mixture was incubated in a 100 °C water bath for 15 min. After cooling to room temperature, the volume was brought to 25 mL with distilled water and allowed to stand for 10–15 min. The absorbance was finally measured at 570 nm wavelength using a 10 mm cuvette. Theaflavins, thearubigins, and theabrownins were systematically analyzed: a tea sample (3 g) was added to 125 mL of boiling water and extracted in a 100 °C water bath for 10 min. The mixture was filtered under suction, and the filtrate was reserved for subsequent use. Then, 25 mL of the prepared solution was transferred to a 100 mL separatory funnel, where it was mixed with 25 mL of ethyl acetate and vigorously shaken for 5 min for extraction. After phase separation, 2 mL of the upper organic layer was collected and combined with 95% ethanol to prepare solution A. From the remaining organic phase, 15 mL was taken and mixed with 15 mL of 2.5% NaHCO_3_ aqueous solution to obtain solution C. Another 2 mL aliquot of the organic layer was combined with 2 mL of saturated oxalic acid solution and 6 mL of distilled water to prepare solution D. In a parallel procedure, 25 mL of the initial tea solution was extracted with 25 mL of n-butanol by shaking for 3 min. Following phase separation, 2 mL of the aqueous layer was collected and reacted with 2 mL of saturated oxalic acid solution plus 6 mL of distilled water to yield solution B. The absorbance values of solutions A, B, C, and D were measured at 380 nm wavelength using a 10 mm cuvette. The content of soluble sugar was determined by anthrone–sulfuric acid colorimetry: a tea sample (1 g) was mixed with 40 mL of 80% ethanol and extracted in a 100 °C water bath for 1 h. The mixture was filtered under suction, and the volume was adjusted to 100 mL with distilled water. From the resulting solution, 1 mL was aliquoted and mixed with 4 mL of anthrone–sulfuric acid reagent. The mixture was heated in a 100 °C water bath for 7 min. After cooling to room temperature, the absorbance was measured at 620 nm wavelength using a 10 mm cuvette.

Catechin and purine alkaloids were determined by HPLC under the following conditions: C18 column; Diode Array Detector (DAD); column temperature: 35 °C, injection volume: 10 μL; mobile phase A was methanol; B was 0.1% trifluoroacetic acid solution; flow rate: 1 mL/min; detection wavelength: 278 nm: elution gradient: 0~2 min, 2%A; 2~8 min, 8%A; 8~30 min, 10%A; 30~35 min, 20%A; 35~45 min, 25%A; 45~55 min, 30%A; 55~60 min, 35%A.

The calculation formula is as follows:

$$\mathrm{polyphenols}\;(\mathrm{mg}/g)=\frac{A\times V1\times d}{\mathrm{SLOPE}\times m\times {w\times 10}^{6}}\times 1000$$

$$\mathrm{Flavones}\;(\mathrm{mg}/g)=\frac{A\times 320}{1000}\times \frac{V1}{V2\times m}$$

$$\mathrm{total}\;\mathrm{free}\;\mathrm{amino}\;\mathrm{acids}\;(\mathrm{mg}/g)=\frac{\frac{c}{1000}\times \frac{V1}{V2}}{m\times w}\times 100$$

$$\mathrm{theaflavins}\;(\mathrm{mg}/g)=\frac{A_{C}\times 2.25}{m\times w}\times 1000$$

$$\mathrm{thearubigins}\;(\mathrm{mg}/g)=\frac{7.06\times (2A_{D}+2A_{A}-A_{C}-2A_{B})}{m\times w}\times 1000$$

$$\mathrm{theabrownins}\;(\mathrm{mg}/g)=\frac{7.06\times 2A_{B}}{m\times w}\times 1000$$

$$\mathrm{soluble}\;\mathrm{sugar}\;(\mathrm{mg}/g)=\frac{\frac{c}{1000}\times V1}{m\times w}\times 1000$$

$$\mathrm{catechin}\;(\mathrm{mg}/g)=\frac{c\times V1}{m\times w}\times 1000$$

$$\mathrm{purine}\;\mathrm{alkaloids}\;(\mathrm{mg}/g)=\frac{c\times V1}{m\times w}\times 1000$$

A: Absorbance of sample test solution;

V1: Total volume of sample (mL);

V2: Volume of measured liquid (mL);

m: Sample quality (g);

W: Dry weight (%);

C: Concentration calculated based on standard curve (ug/mL);

SLOPE: Slope of gallic acid standard curve.

### 2.5. Determination of Volatile Substances

The determination was performed by GC-MS (Agilent Technologies Co., LTD., Santa Clara, CA, USA).

Tea sample pretreatment: Volatile components of the tea sample were extracted by headspace solid-phase microextraction: 2 g of tea powder was weighed and transferred immediately to a 40 mL headspace vial, containing 2 mL NaCl saturated solution, and 20 μL 2-phenethyl acetate (5 mg/L) was added as an internal standard (IS). The vial was sealed with two layers of tin foil and preheated at 80 °C for 40 min. DVB/CAR/PDMS fiber (inner diameter: 50/30 μm; length: 2 cm) was inserted into the headspace bottle, and the fiber head was rotated to extract at 80 °C for 40 min. After extraction, the extracted fiber was put into the entrance of the gas chromatograph and analyzed at 250 °C for 3 min.

Analytical conditions: HP-5MS capillary column (30 m × 0.25 mm × 0.25 μm); the GC/MS capillary was supported by high-purity helium gas with a flow rate of 1.0 mL/min and no shunt mode. Injection volume: 1 μL. Heating procedure: 50 °C for the first 1 min, then 220 °C at a speed of 5 °C/min for 5 min. Mass spectra were scanned in the range of 30–350 m/z, and the solvent delay time was 4 min. The mass spectrum ion source was set to 230 °C with an electron energy of 70 eV.

Qualitative analysis: The mass spectrometry data obtained from the GC-MS analysis were compared against the National Standard Institute of Standards and Technology (NIST17.L) database combined with Automated Mass Spectral Deconvolution and Identification System (AMDIS) deconvolution. Compounds with a similarity match >80% were tentatively identified. Additionally, the retention index (RI) of unknown chromatographic peaks was calculated using C7~C40 n-alkanes and cross-referenced with the RI values of compounds in the NIST17 database, RI values reported in the literature, authentic standards, and aroma characteristics for further confirmation.

Quantitative analysis: The semi-quantitative analysis of the identified aroma components was conducted using the internal standard method. The relative content of each compound was determined by calculating the ratio of its peak area to the total peak area of the internal standard (IS). Each tea sample was analyzed in triplicate, and the mean value along with the relative standard deviation (RSD) was calculated. The relative concentrations of the volatile components were calculated using the following formula:

Relative concentration (ug/g)=[(Peak area of target/Peak area of IS)×0.1 ug]/(2 g)

and the sum of all volatile components was calculated as total volatiles of each sample.

### 2.6. Determination of Non-Volatile Substances

The determination was performed by UHPLC-Q-Exactive/MS (Agilent Technologies Co., LTD, Santa Clara, CA, USA).

Tea sample pretreatment: In total, 20 mg tea powder was mixed with 1000 μL extract solution (methanol/water = 3:1 (*V*/*V*), containing an internal standard mixture of isotope labels). After grinding at 35 Hz for 4 min, ultrasonic treatment was performed for 5 min (ice water bath), repeated 2–3 times, and left at −40 °C for 1 h. The sample was centrifuged at 4 °C, 12,000 rpm (RCF = 13,800 (g); R = 8.6 cm), for 15 min. Finally, the supernatant was taken into the sample bottle and tested.

Analytical conditions: The target compounds were separated using a Water ACQUITY UPLC HSS T3 (2.1 mm × 100 mm, 1.8 μm) liquid chromatographic column. Phase A was aqueous, containing 5 mmol/L ammonium acetate and 5 mmol/L acetic acid, and phase B was acetonitrile. The temperature of the automatic sampler was 4 °C and the injection volume was 2 μL. The Orbitrap Exploris 120 mass spectrometer acquired primary and secondary mass spectrometry data under the control of Xcalibur software (version 4.4, Thermo, Waltham, MA, USA). The acquisition software (4.4) continuously evaluates the full-scan MS spectrum, with ESI source conditions set as follows: the sheath gas flow rate was 50 Arb, the auxiliary gas flow rate was 15 Arb, the capillary temperature was 320 °C, the full MS resolution was 60,000, the MS/MS resolution was 15,000, the energy was 10/30/60 in NCE mode, and the spray voltage was 3.8 kV (positive) or −3.4 kV (negative).

### 2.7. Sensory Review Method

Ten professionals certified as Advanced Tea Evaluators were invited to conduct a sensory evaluation of tea samples according to the provisions of the GB/T 23776-2018 [22] Sensory Evaluation Method for dark tea (loose tea). The main procedures were carried out as follows: a tea sample (3 g) was weighed with a tea-to-water ratio (mass/volume) of 1:50 and placed in the designated tasting cup. The cup was filled with boiling water, covered, and steeped for 2 min. The tea liquor was then decanted at a consistent speed in sequential order into tasting bowls. The liquor color was evaluated, the aroma of the wet leaves in the cup was assessed, and the taste was sampled. Subsequently, a second infusion was prepared with a steeping time of 5 min. The tea liquor was again decanted, and evaluations were conducted for liquor color, aroma, taste, and wet leaf appearance. The appearance (20%), soup color (15%), aroma (25%), taste (30%), and leaf base (10%) were weighted accordingly.

### 2.8. Cell Experiment

Freeze-dried tea sample preparation and cell preparation: The tea samples were frozen at −80 °C for 12 h prior to lyophilization using a Christ Alpha 1–4 LDplus freeze-dryer (Martin Christ GmbH, Wilzheim, Germany), with the condenser maintained at −85 °C, and dried at 0.1 mbar for 48 h. The freeze-dried tea sample was ground into powder and passed through a 60-mesh sieve. The ratio of tea to water (g/mL) was 1:100, and the extraction temperature was 100 °C for 20 min. After filtration, the concentrated green leaves were freeze-dried and refrigerated at 4 °C. The cells used in the experiment were a human hepatocellular liver carcinoma cell line (HepG2). The frozen cells were removed from liquid nitrogen, thawed in a water bath of 37~40 °C for 1 min, and quickly moved to a super-clean table; then 6 mL complete medium was added, the mixture was centrifuged at 1000 r/min for 5 min, the supernatant was discarded, and 8 mL complete medium was added. The cells were transferred to a culture bottle and incubated at 37 °C for 3 days. Microscopically, the cells grew to 80% for subculture. The old medium was discarded, the cells were rinsed twice with PBS, and 1 mL pancreatin was added for digestion at 37 °C for 2–5 min until the cells became round and detached. Then, 3 mL complete medium was added to stop digestion, the cells were gently resuspended, and the concentration was adjusted on a hemocytometer for further experiments.

Oleic acid toxicity test: A cell suspension was inoculated into 96-well plates with a density of 6 × 10^3^ cells/well and a volume of 100 μL per well. After 24 h incubation at 37 °C, the supernatant was discarded. The medium with different concentrations of OA was added to the experimental group, while the medium without OA was added to the control group. The final volume of each well was 100 μL. After 24 h, 20 μL (5 mg/mL) MTT solution was added to each well. After 4 h, the supernatant was discarded, and 100 μL DMSO was added to each well. The absorbance was measured at 570 nm after shaking with an automatic enzyme marker.

Toxicity test of tea extract: A cell suspension was inoculated into 96-well plates with a density of 6 × 10^3^ cells/well and a volume of 100 μL per well. After 24 h incubation at 37 °C, the supernatant was discarded. The medium with different concentrations of tea extract was added to the experimental group, while the medium without tea extract was added to the control group. The final volume of each well was 100 μL. After 24 h, 20 μL 5 mg/mL MTT solution was added to each well, and after 4 h, the supernatant was discarded and 100 μL DMSO was added to each well, and the absorbance was measured at 570 nm after shaking with an automatic enzyme label.

Hyperlipidemia model establishment: A cell suspension was inoculated into a black 96-well plate at a density of 4 × 10 cells/well and a volume of 100 μL per well. After 24 h incubation at 37 °C, the supernatant was discarded. The test group received non-toxic OA medium, while the control received OA-free medium. The final volume of each well was 100 μL. After 24 h, the supernatant was discarded, 100 μL PBS was added to each well, and 100 μL of 0.8 μg/mL NR solution (20% DMSO + 80% PBS) was added to each well. The solution was stained at 37 °C for 6 min at an excitation wavelength of 485 nm. The fluorescence intensity was detected at 538 nm.

Effect of tea extract on lipid production in HepG2 cells: A cell suspension was inoculated into black 96-well plates at a density of 4 × 10 cells/well and a volume of 100 μL per well. After 24 h incubation at 37 °C, the supernatant was discarded. The test group received non-toxic OA medium, while the control received OA-free medium. The final volume of each well was 100 μL. After 24 h, the supernatant was discarded and tea extract with non-toxic concentration was added to each well. After 24 h, the supernatant was discarded and 100 μL PBS was added to each well, and 100 μL of 0.8 μg/mL NR solution (20% DMSO + 80% PBS) was added to each well. The fluorescence intensity was detected at an excitation wavelength of 485 nm and an emission wavelength of 538 nm after staining at 37 °C for 6 min under the condition of avoiding light.

### 2.9. Data Analysis

SPSS 19.0 and Excle 2018 were used for data processing and table making. Line charts were created with Origin 2018; Prism 8.0 was used to draw bar charts; heat maps were created with TBtools-II2.225. Principal component analysis and volcano map drawing were generated using the Baiqu online platform (www.lims2.com, accessed on 19 December 2024).

## 3. Results and Discussion

### 3.1. Results of Isolation and Identification of Monascus

**Figure 1:**
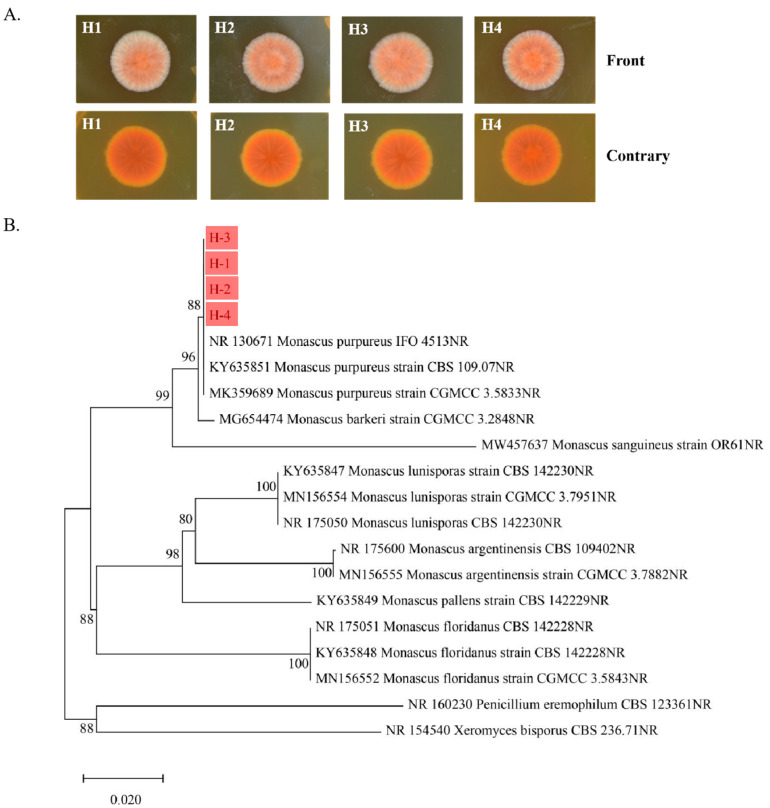
Monascus colony morphology (**A**) and phylogenetic tree of monascus based on DNA sequence analysis (**B**). The red parts represent the four types of monascus that we identified.

Four characteristic strains of monascus were isolated from red yeast rice and named H-1, H-2, H-3, and H-4. A single colony was inoculated in MEA medium by the spot seeding method and cultured at 28 °C for 10 days to observe the characteristics of the colony (Figure 1A). The four strains all produced red pigment, and colonies were membranous, felt-like, with little folds or radiating, which were the typical characteristics of monasgillus.

The four strains were sequenced and compared with other strains of monascus to construct the evolutionary tree. As shown in Figure 1B, the 14 known strains of monascus form their own relatively independent branches in the evolutionary tree, and H-1, H-2, H-3, and H-4 are all clustered in one branch with 3 strains of monascus (IFO 4513, CBS 109.07, and CGMCC 3.5833). This branch has a high cladic confidence and is adjacent to the monascus barkeri strain CGMCC 3.2848, entry number MG654474. Combined with colony characteristics and biological identification, H-1, H-2, H-3, and H-4 were presumed to be monascus purpureus.

### 3.2. Sensory Evaluation of UT and FT

The sensory evaluation results of UT and FT are shown in Table 1. UT has a loose shape, the soup color is bright orange; there is a floral display in terms of aroma; the taste is fresh and pure; the astringent taste is more obvious; and there is a soft and bright leaf bottom. FT’s is appearance bluish brown, with white mold spots; the soup color is deeper than that of UT; there is a baking aroma as well as a mushroom aroma; the tea has a mellow and slightly bitter taste, and a soft and bright leaf bottom.

### 3.3. Comparative Analysis of Volatile Substances in UT and FT

**Figure 2:**
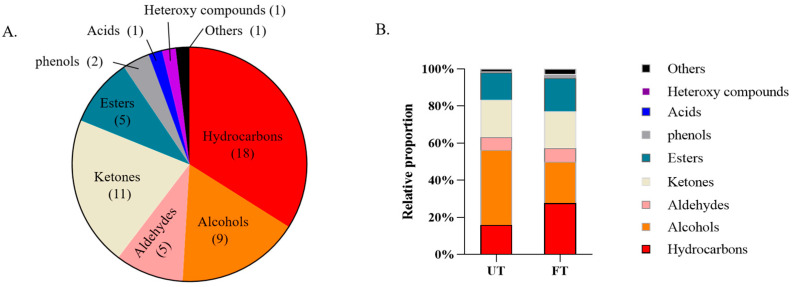
Aroma substances of UT and FT. (**A**) Classification diagram of aroma substances detected in total. (**B**) Proportion of aroma substance types of UT and FT.

In order to explore the aroma components of FT and UT, we determined them by GC-MS. A total of 53 components were detected in the two tea samples through spectrum library search and mass spectrometry. As shown in Figure 2A, the 53 aroma substances are classified into nine categories, of which the largest number is hydrocarbon oxides, followed by ketones, alcohols, aldehydes, esters, phenols, acids, heterooxygen compounds, and other substances (11, 9, 5, 5, 2, 1, 1, and 1). By calculating the proportion of different aroma types in UT and FT, we found that UT was dominated by alcohols and FT was dominated by hydrocarbons (Figure 2B). Studies have shown that dark tea fermentation produces more phenolic acids and terpenes, and the tea samples after monascus fermentation are mainly terpenes [16]. Terpenes belong to hydrocarbon oxides, and the aroma substances of FT in this study are mainly hydrocarbon oxides, which is consistent with previous studies.

**Figure 3:**
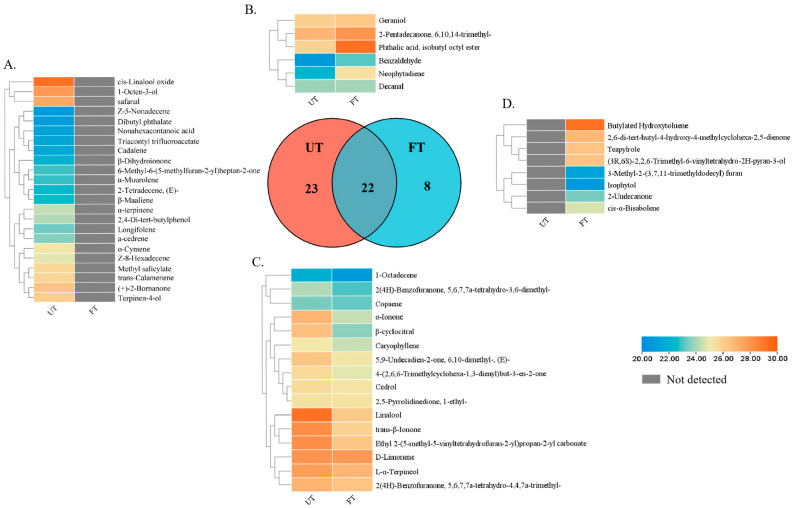
Changes in aroma substances in UT and FT. (**A**) Aroma substances detected by UT. (**B**) Aroma substances with increased content in FT. (**C**) Aroma substances with reduced content in FT. (**D**) Aroma substances detected in FT.

Further analysis showed that 23 aromatic substances were not detected in FT, such as methyl salicylate and 1-Octen-3-ol. All of these substances are floral, fragrant, or fruity (Figure 3A). Eight new substances were detected in FT, which were manifested as baking aroma and wood aroma (Figure 3D). Among the 22 substances shared by UT and FT, the content of 6 substances increased in FT and that of 16 substances decreased in FT (Figure 3B,C).

**Figure 4:**
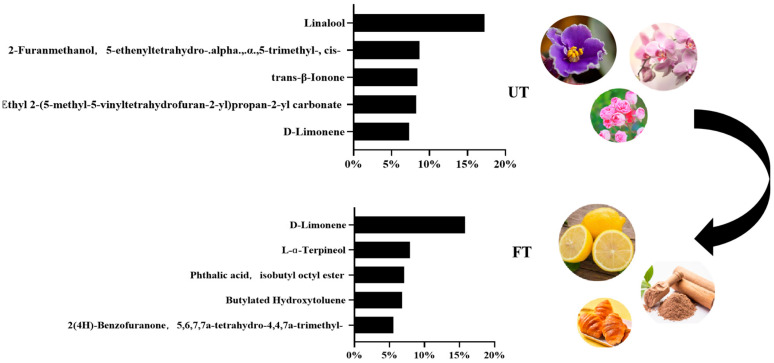
Aroma ratio of UT and FT.

The characteristic aroma substances of FT and UT were screened by analyzing the composition and proportion of aroma substances. The aroma substances detected in the two tea samples and their relative contents are shown in Table S1. During the processing of tea or in the finished tea, some unpleasant aromas do indeed occur, such as aldehydes, sulfides, and low-level fatty acids, which may produce grassy, aged, or rancidity flavors. Most aroma substances exhibit a dual nature—they present a pleasant floral and fruity scent at low concentrations, but an unpleasant odor when the concentration is too high or the proportion is too large. For instance, Benzaldehyde exhibits the floral and fruity aroma of sweet almonds at low concentrations, while at high concentrations, it may reveal unpleasant odors such as bitterness and medicinal notes. The proportions of Benzaldehyde in UT and FT were 0.40% and 1.67%, respectively, which were relatively low. Its effect on the aroma of tea samples was pleasant. We screened the top five aroma substances with the highest proportion. As shown in Figure 4, the main aroma substances in UT were linalool, 2-Furanmethanol, 5-ethenyltetrahydro-a, 5-trimethyl-, cis-, trans-β-Ionone, Ethyl 2-(5-methyl-5-vinyltetrahydrofuran-2-yl)propan-2-yl carbonate, and D-Limonene, with floral notes, such as violet and orchid. The main aroma substances in FT were D-Limonene, L-Alpha Terpineol, Phthalic acid, isobutyl octyl ester, Butylated Hydroxytoluene, and 2(4H)-Benzofuranone, 5, 6, 7, 7 a-Tetrahydro-4, 4, 7a-trimethyl-, manifested as lemon, toast, and wood.

Previous studies have reported that the addition of microorganisms can change the aroma of tea [23]. The fermentation of *Aspergillus niger* produces camphor flavor in tea, the fermentation of yeast produces a distinctly stale aroma, and the solid fermentation of tea with rhizopus has a unique sweet aroma. Tian et al. [16] showed that after monascus fermentation, 1, 2-dimethoxybenzene, 1, 2, 3-trimethoxybenzene, (E)-linalool oxide (pyranoid), methyl salicylate, linalool, β-ionone, and β-damascenone were the main characteristic aroma substances, manifested as stale and woody aromas [16]. In this study, after the mixed inoculation of various monascus species, the flower fragrance of UT was transformed into the lemon and wood fragrance of FT, with some old fragrance, which was consistent with previous studies.

### 3.4. Comparative Analysis of Non-Volatile Substances in UT and FT

Studies have shown that monascus can produce a large number of secondary metabolites, such as monascus pigment, monacolin K, and GABA [24]. In order to investigate the changes in secondary metabolites in Pu-erh tea inoculated with monascus, the main biochemical components and metabolomics of FT and UT were determined and analyzed.

#### 3.4.1. Main Biochemical Components of UT and FT

**Figure 5:**
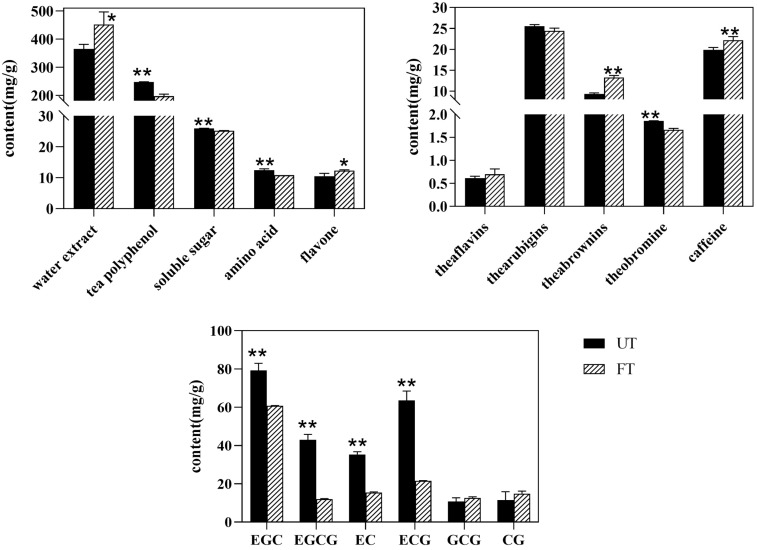
The main biochemical components of UT and FT. * indicates a significant difference: * 0.01 ≤ *P* < 0.05 and ** *P* < 0.01.

The purified strains were mixed into Pu-erh raw tea in equal proportion for fermentation, and the tea sample (FT) after fermentation was obtained. The main biochemical components of unfermented Pu-erh tea (UT) and fermented Pu-erh tea (FT) were determined, and the results are shown in Figure 5. The water extract in FT (452.22 mg/g) was significantly higher than that in UT (364.44 mg/g), increasing by 19.41%. Similarly, flavones in FT (12.32 mg/g) were also significantly higher than in UT (10.54 mg/g), increasing by 14.47%. On the other hand, the total amount of tea polyphenols, free amino acids, and soluble sugars decreased in FT. Among them, the content of tea polyphenols in FT (197.24 mg/g) was significantly lower than that in UT (246.78 mg/g), with a reduction of 20.07%. Furthermore, catechins (8 monomers), the main substances of tea polyphenols, were determined. The cis-catechins contents (EGCG, EGC, EC, and ECG) of FT were 12.62 mg/g, 77.34 mg/g, 35.40 mg/g, and 63.65 mg/g, respectively, and the cis-catechins contents (EGCG, EGC, EC, and ECG) of UT were 43.02 mg/g, 60.87 mg/g, 15.42 mg/g, and 21.55 mg/g, respectively. The results showed that cis-catechins (EGCG, EGC, EC, and ECG) in FT were significantly lower than UT. The trans-catechins contents (GCG and CG) of FT were 12.61 mg/g and 14.88 mg/g, respectively, and those of UT were 10.76 mg/g and 11.57 mg/g, respectively. The results showed that trans-catechins (GCG and CG) in FT were higher than in UT. C and CG were not detected in the two tea samples. There were also differences in the theaflavin, thearubigin, and theabrownin contents among the two tea samples, and the theabrownin content of FT (13.24 mg/g) was significantly higher than that of UT (9.29 mg/g), increased by 29.82%. In addition, we also determined the purine alkaloid content of two tea samples, and the results showed that the theobromine content of FT (1.66 mg/g) was significantly lower than that of UT (1.85 mg/g), decreased by 10.41%, while the caffeine content of FT (22.18 mg/g) was significantly higher than that of UT (19.82 mg/g), increased by 10.67%.

The results indicated that there were significant differences in the biochemical composition and sensory evaluation of UT and FT. Monascus can produce a large amount of protease, esterase, and amylase and has a strong saccharification and esterification power, which can promote the conversion of substances contained in tea [25]. The increase in water extract content in FT was attributed to the decomposition of originally water-insoluble substances by monascus, resulting in the formation of new soluble components. This process enhances the richness and mellowness of the tea flavor [26]. Additionally, polyphenols were degraded in the fermentation process, and theaflavins, thearubigins, theabrownins, and other products of oxidative polymerization were increased. Consequently, a large amount of theabrownin accumulates in FT, resulting in a darker tea soup color, which is consistent with other fermented Pu-erh tea [27]. The flavonoid content of FT was increased, which was consistent with the previous results of the mixed fermentation of Pu-erh raw tea with monascus and yeast. Interestingly, the theobromine content in FT was significantly lower than that in UT, and the caffeine content was significantly higher than that in UT, while there was no significant difference between theobromine and caffeine in tea before and after monascus fermentation in previous studies [17]. Theobromine is a precursor to caffeine synthesis. Caffeine production is accompanied by theobromine consumption, which may be due to the action of various monascus fungi, which enhances the ability of theobromine metabolism to caffeine, resulting in the accumulation of more caffeine in FT, which makes the tea taste bitter. Theabrownins, flavonoids, and caffeine all have more physiological activities, such as anti-inflammatory, antioxidant, lipid-lowering, and nerve-excitatory functions [28,29,30]. Research has demonstrated that these bioactive components contribute to the substantial health-promoting properties observed in FT.

#### 3.4.2. Overall Changes and Screening of Characteristic Substances in UT and FT

**Figure 6:**
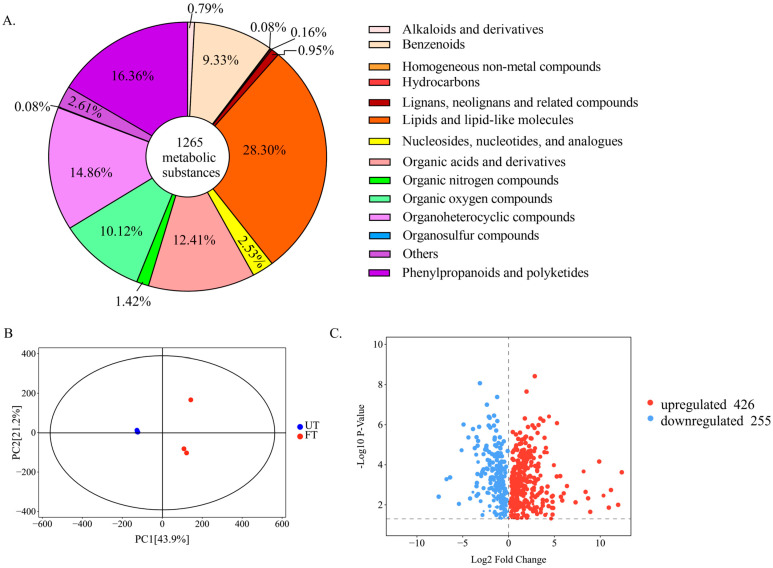
Holistic analysis of metabolic substances. (**A**) Secondary classification and proportion of metabolic substances detected by UT and FT. (**B**) PCA plot of tea samples. (**C**) Volcano plot of different metabolites.

More than 800 metabolites of monasgillus are known, including amino acids and amines, organic acids, sugars and sugar alcohols, esters, alkanes and polyketoids, ethanol, chlorogenic acid, glycholate, vitamins, coenzymes, and nucleotides [31,32,33]. A total of 1265 substances were detected in the two tea samples using metabolomic assays, which were classified into two classes and copolymerized into 14 types of substances, as shown in Figure 6A. The largest proportions were those of lipids and lipid-like molecules (28.30%), phenylpropanoids and polyketides (16.36%), and organoheterocyclic compounds (14.86%). By PCA (principal component analysis), the two samples were clearly distinguished (Figure 6B). After screening the detected substances, the screening criteria were |FC| ≥ 2 and VIP > 1. A total of 681 differential substances were identified, of which 426 substances were upregulated in FT and 255 substances were downregulated in FT (Figure 6C).

**Figure 7:**
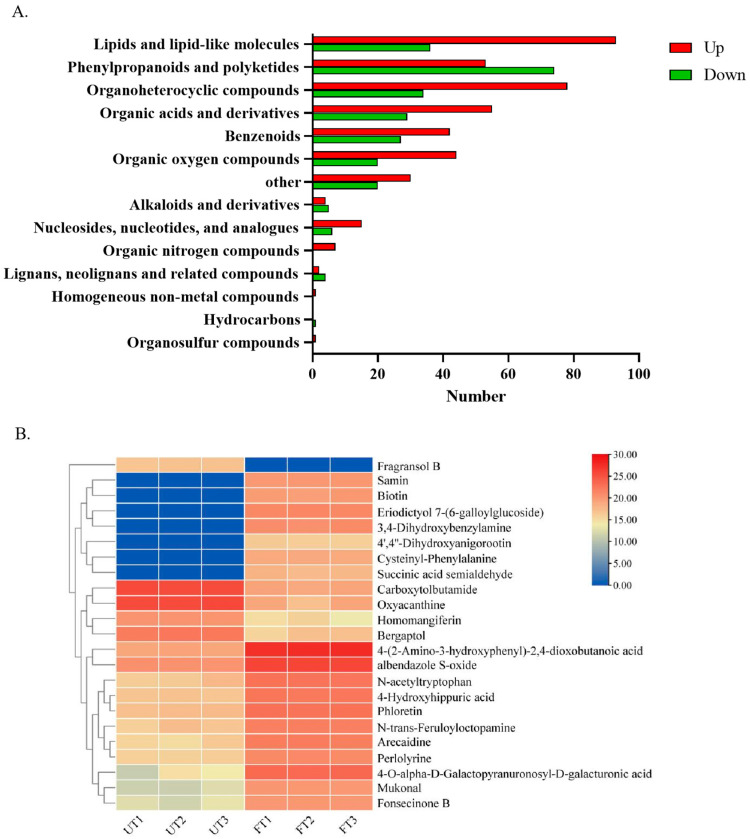
Differential metabolite enrichment analysis. (**A**) Secondary classification histogram of differential metabolites. (**B**) Heat maps of characteristic differential metabolites of UT and FT (|FC| > 5).

The different substances were classified and analyzed in secondary classification. The differences were mainly concentrated in lipids and lips-like molecules, phenylpropanoids and polyketides, organoheterocyclic compounds, organic acids and compound derivatives, and benzenoids. Among them, the substances upregulated by FT are mainly lipids and lipid-like molecules, while the substances downregulated are mainly phenylpropanoids and polyketides (Figure 7A).

In order to obtain the characteristic metabolites of FT, 681 different substances were re-screened, and the screening condition was |FC| value ≥ 5. As shown in Figure 7B, a total of 23 different substances were obtained, of which 18 were upregulated and 5 were downregulated in FT. Of the 18 substances upregulated, 7 were new substances (Samin, Biotin, Eriodictyol 7-(6-galloylglucoside), 3, 4-dihydroxybenzylamine, 4′, 4″-Dihydroxyanigorootin, Cysteinyl-Phenylalanine, and Succinic acid semialdehyde). These substances may be produced under the action of monascus *aspergillus*. They can be used as the characteristic metabolites of FT. These substances are mainly fatty acids, amino acids, and flavonoids. The main metabolites of monascus were monascus pigments, citrinin, monacolin K, and lovastatin. These are all polyketones that share the same synthetic precursors (acetyl-CoA and propionyl-CoA) and similar synthetic pathways [34]. Monascus pigments, citrinin, monacolin K, and lovastatin were not detected in UT or FT. Deng et al. detected only a very small amount of lovastatin (0.011%) and no other substances after fermenting Pu-erh raw tea with purpurus monascus [17]. Acetyl-CoA is a precursor for the synthesis of many secondary metabolites. The synthesis of both polyketone compounds and fatty acid substances requires acetyl-CoA, and there is a competitive relationship between the two [35]. In addition, succinic acid is an intermediate product of the tricarboxylic acid cycle. The exogenous addition of succinic acid can significantly downregulate the expression of genes related to fatty acid biosynthesis in monascus, while upregulating the expression of genes related to pyruvate metabolism, thereby increasing the yield of monascus pigments [36]. In this study, no polyketone compounds were detected. However, we found that the content of succinic acid in FT was significantly downregulated, while the content of fatty acids was significantly increased, which may competitively regulate the biosynthetic metabolic pathway of acetyl-CoA, resulting in extremely low levels of monascus pigments.

#### 3.4.3. Changes in Flavor-Related Substances of UT and FT

**Figure 8:**
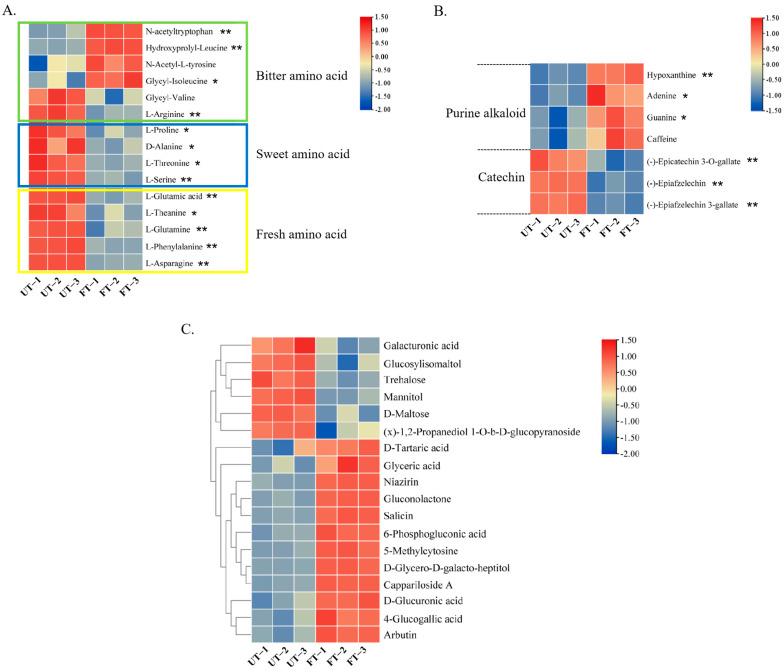
Heat maps of UT and FT flavor-related metabolites. (**A**) Amino acids. (**B**) Purine alkaloids and catechins. (**C**) Sugar substances. * indicates a significant difference: * 0.01 ≤ *P* < 0.05 and ** *P* < 0.01.

During the fermentation process of Pu-erh tea, a series of intense chemical changes occur, resulting in significant differences in flavor between the fermented and unfermented tea leaves. To investigate the impact of monascus fermentation on the flavor of tea, we conducted a comparative analysis of the main flavor compounds in tea: amino acids, catechins, purine alkaloids, and soluble sugars. There are primarily 20 types of amino acids in tea, which can be categorized into umami, bitter, and sweet amino acids [37]. As shown in Figure 8A, all sweet and umami amino acids in FT are significantly lower than in UT, while three bitter amino acids are significantly higher than in UT, resulting in a bitter flavor and lower freshness in FT. Purine alkaloids are the main compounds contributing to the bitterness of the tea infusion, while catechins are the primary compounds responsible for its astringency [38]. As illustrated in Figure 8B, the purine alkaloids in FT are all upregulated, whereas catechins show a downregulation, leading to a bitter flavor with reduced astringency in FT. Sugars are important factors in forming the sweetness and richness of the tea infusion [39]. As depicted in Figure 8C, there are significant differences in the content of 18 sugar compounds between the two tea samples, with 12 types significantly upregulated in FT. The synergistic effects of these flavor compounds—amino acids, purine alkaloids, catechins, and sugars—result in a rich and slightly bitter taste in FT.

Amino acids and caffeine are not only the main flavor components, but also the nitrogen sources of microorganisms in the fermentation process of Pu-erh tea. Microorganisms degrade three large amino acids in tea—theanine, glutamic acid, and asparagine—as nitrogen sources for their growth and reproduction, and synthesize amino acids with low content in tea through microbial metabolic activities. We found that the contents of theanine, glutamic acid, and asparagine decreased significantly in FT, while the content of caffeine increased. Therefore, the nitrogen source of monascus was mainly amino acids. During the fermentation of Pu-erh tea, sugars, on the one hand, provide carbon source for microorganisms; on the other hand, extracellular cellulase is secreted to gradually decompose the abundant cellulose in tea into soluble sugar, forming a dynamic equilibrium result. The changes in sugar substances in our study are also consistent with previous studies. Some sugar substances are degraded, while the content of most sugar substances have increased, which increases the thickness and richness of tea soup to a certain extent.

#### 3.4.4. Changes in Potential Lipid-Lowering Substances of UT and FT

**Figure 9:**
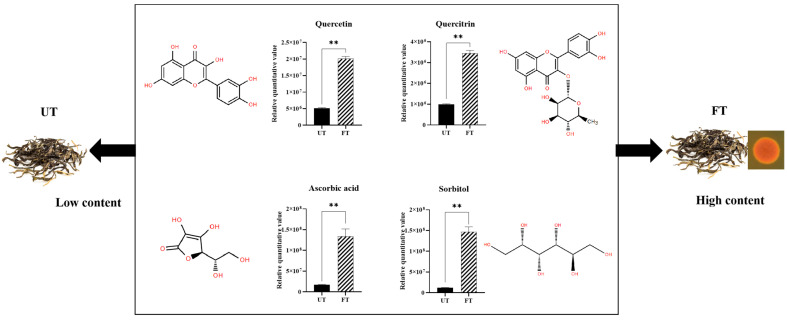
Relative contents of lipid-lowering substances in UT and FT. * indicates a significant difference: ** *P* < 0.01.

A series of foods fermented by monascus, such as red yeast rice, red yeast cheese, and red yeast soy sauce, produce active substances like GABA, lovastatin, ergosterol, and red yeast polysaccharides, which confer benefits such as lowering blood lipids, reducing blood pressure, lowering blood sugar, and combating fatigue [40,41]. Tea contains a large number of active substances, such as flavonoids and amino acids [42]. Our quantitative analysis of the functional substances in the two tea samples revealed that substances with effects on lowering blood lipids and blood pressure were significantly upregulated in FT. As shown in Figure 9, the relative contents of active substances with lipid-lowering effects such as quercetin, quercitrin, ascorbic acid, and sorbitol in FT were higher than those in UT, and increased 83.09%, 81.73%, 89.86%, and 92.76%, respectively. Quercetin and quercitrin are widely occurring flavonoid monomer compounds found in plants, possessing various pharmacological effects such as antioxidant, anti-tumor, blood sugar-lowering, and blood lipid-lowering properties [43]. Research by Cao et al. indicates that quercetin can improve high-fat-diet-induced NAFLD through AMPK-mediated mitochondrial autophagy [44]. Ascorbic acid enhances the body’s resistance and has antioxidant effects [45]. Sorbitol can reduce calories and sweetness, making it friendly for diabetic patients. The content of these substances in FT has significantly increased, enhancing the application value of FT.

### 3.5. Study on Lipid-Lowering Function of UT and FT In Vitro

**Figure 10:**
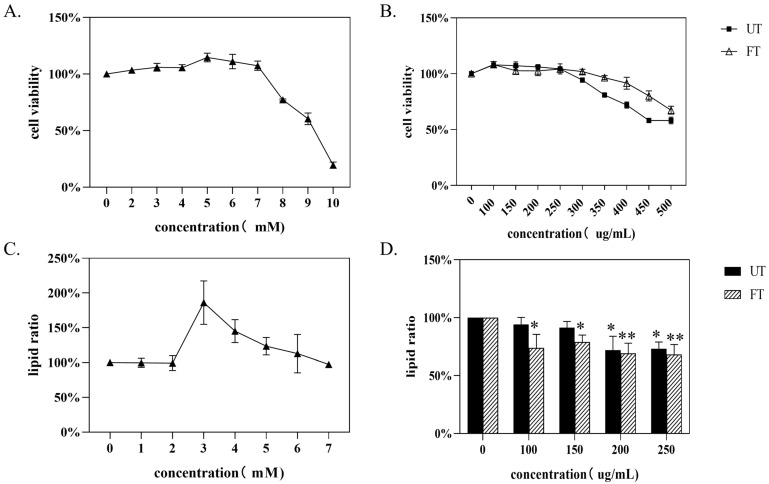
Effects of UT and FT on lipid lowering of cells. (**A**) Effect of OA concentration on survival rate of HepG2. (**B**) Effects of different concentrations of UT and FT extracts on survival rate of HepG2. (**C**) Effect of OA on lipid formation in HepG2. (**D**) Effects of UT and FT on lipid formation in HepG2. * indicates a significant difference: * 0.01 ≤ *P* < 0.05 and ** *P* < 0.01.

Studies have shown that the lipid-lowering function of fermented tea leaves is higher than that of unfermented tea leaves [46]. Deng et al. [17] found that monascus-fermented Pu-erh tea had better hypolipidemic and anti-atherosclerotic effects than Pu-erh raw tea. In the above analysis, we also found that substances with lipid-lowering effects in FT, such as quercetin and quercitrin, were significantly higher than those in UT. Therefore, we studied the lipid-lowering function of UT and FT. First, we applied different concentrations of oleic acid (OA) to HepG2 cells to obtain the optimal oleic acid concentration for establishing a high-fat HepG2 cell model. As shown in Figure 10A, toxicity began to manifest when OA = 7 mM/L, at which point cell viability was 109.3%. When OA = 10 mM/L, the cell viability was only 19.8%. Therefore, the oleic acid concentration applied by the model does not exceed 7 mM/L. On this basis, we measured the intracellular lipid content administered with different concentrations of OA, and the results shown in Figure 10C showed that when OA = 3 mM/L, the intracellular lipid content reached a maximum of 113.5%, so this concentration was selected as the cell high-lipid modeling concentration.

Subsequently, we conducted toxicity tests on tea extracts. As shown in Figure 10B, when the concentration of the tea sample extract is ≤250 μg/mL, neither UT nor FT exhibit any toxic effects on the cells. At an extract concentration of 300 μg/mL, FT also shows no toxicity to the cells, with a cell survival rate of 101.9%. However, the cell survival rate for UT slightly decreases to 94.36%. When the extract concentration exceeds 300 μg/mL, both UT and FT exhibit toxicity to the cells. Therefore, the concentration of the tea sample administered to the cells should be less than 300 μg/mL. Based on this, we applied different concentrations of UT and FT to high-fat HepG2 cells, with the results shown in Figure 10D. The application of UT and FT at concentrations of 100 μg/mL to 300 μg/mL is effective in reducing lipid levels in the cells. Among them, UT shows significant effects on lipid reduction at concentrations greater than 200 μg/mL, while FT demonstrates significant effects at concentrations greater than 100 μg/mL. Furthermore, as the concentration increases, the lipid-reducing effect becomes extremely significant, indicating that FT has a better lipid-reducing effect than UT.

## 4. Conclusions

The quality and functional properties of monascus-fermented Pu-erh raw tea (FT) exhibit significant differences compared to unfermented Pu-erh raw tea (UT). Sensory evaluation revealed distinct variations in aroma and taste profiles: FT demonstrated a richer and slightly more bitter taste than UT, accompanied by a pronounced fungal aroma. To elucidate the underlying mechanisms of these differences, multi-omics analysis was employed. In terms of aroma composition, UT was characterized by floral notes, primarily attributed to linalool, 2-Furanmethanol, 5-ethenyltetrahydro-a, 5-trimethyl-, cis-, trans-β-Ionone, Ethyl 2-(5-methyl-5-vinyltetrahydrofuran-2-yl)propan-2-yl carbonate, and D-Limonene. In contrast, FT exhibited citrusy, roasted, and woody aromas, dominated by D-Limonene, L-α-Terpineol, Phthalic acid, isobutyl octyl ester, Butylated Hydroxytoluene, and 2(4H)-Benzofuranone, 5, 6, 7, 7a-tetrahydro-4, 4, 7a-trimethyl-. In terms of taste composition, FT contained higher levels of bitter amino acids and caffeine, a lower catechin content, and an abundance of sugar-related compounds, contributing to its --+-+-balanced yet mildly bitter and mellow profile. In addition, through the content of the relative peak area, we found that quercetin, quercitrin, ascorbic acid, and sorbitol had significant differences in FT and UT. The relative contents of active substances with lipid-lowering effects such as quercetin, quercitrin, ascorbic acid, and sorbitol in FT were higher than those in UT, and increased 83.09%, 81.73%, 89.86%, and 92.76%, respectively. In vitro cell assays confirmed that FT exhibited superior lipid-lowering efficacy over UT. These findings provide a theoretical foundation for evaluating the characteristic flavor quality and functional benefits of monascus-fermented Pu-erh tea, offering insights for future research and product development.

## Figures and Tables

**Table 1:** Sensory evaluation table of tea samples before and after fermentation.

	Appearance (20%)	Soup Color (15%)	Aroma (25%)	Taste (30%)	Tea Residue (10%)	Gross Score
UT	Loose, many stems	Bright orange	Fragrance of a flower	Pure and refreshing	Bluish yellow, diastolic	88.97
Score	75	93	96	89	93	
FT	Bluish brown with white mildew spots	Orange with a little brightness	Toasty fragrance, floral with bacteria	Peaceful and slightly bitter	Black with yellow green, soft	87.79
Score	80	91	86	91	94	

## Data Availability

The original contributions presented in the study are included in the article, further inquiries can be directed to the corresponding author.

## Supplementary Materials

The following supporting information can be downloaded at: https://www.mdpi.com/article/10.3390/foods14111894/s1. Table S1: The aroma substances and their relative contents were detected in two tea samples.
